# Contrasting breast cancer molecular subtypes across serial tumor progression stages: biological and prognostic implications

**DOI:** 10.18632/oncotarget.5089

**Published:** 2015-09-08

**Authors:** Siker Kimbung, Anikó Kovács, Anna Danielsson, Pär-Ola Bendahl, Kristina Lövgren, Marianne Frostvik Stolt, Nicholas P. Tobin, Linda Lindström, Jonas Bergh, Zakaria Einbeigi, Mårten Fernö, Thomas Hatschek, Ingrid Hedenfalk

**Affiliations:** ^1^ Division of Oncology and Pathology, Department of Clinical Sciences, Lund University, Lund, Sweden; ^2^ CREATE Health Strategic Center for Translational Cancer Research, Lund University, Lund, Sweden; ^3^ Department of Clinical Pathology and Cytology, Sahlgrenska University Hospital, Gothenburg, Sweden; ^4^ Department of Oncology, Institute of Clinical Sciences, Sahlgrenska Academy, University of Gothenburg, Gothenburg, Sweden; ^5^ Department of Oncology and Pathology, Karolinska Institutet and Karolinska University Hospital, Sweden; ^6^ Department of Surgery, University of California at San Francisco (UCSF), San Francisco, CA, USA; ^7^ Department of Biosciences and Nutrition, Karolinska Institutet and University Hospital, Solna, Sweden

**Keywords:** metastatic breast cancer, estrogen receptor, molecular subtype, biomarker conversion, post recurrence mortality

## Abstract

The relevance of the intrinsic subtypes for clinical management of metastatic breast cancer is not comprehensively established. We aimed to evaluate the prevalence and prognostic significance of drifts in tumor molecular subtypes during breast cancer progression. A well-annotated cohort of 304 women with advanced breast cancer was studied. Tissue microarrays of primary tumors and synchronous lymph node metastases were constructed. Conventional biomarkers were centrally assessed and molecular subtypes were assigned following the 2013 St Gallen guidelines. Fine-needle aspirates of asynchronous metastases were transcriptionally profiled and subtyped using PAM50. Discordant expression of individual biomarkers and molecular subtypes was observed during tumor progression. Primary luminal-like tumors were relatively unstable, frequently adopting a more aggressive subtype in the metastases. Notably, loss of ER expression and a luminal to non-luminal subtype conversion was associated with an inferior post-recurrence survival. In addition, ER and molecular subtype assessed at all tumor progression stages were independent prognostic factors for post-recurrence breast cancer mortality in multivariable analyses. Our results demonstrate that drifts in tumor molecular subtypes may occur during tumor progression, conferring adverse consequences on outcome following breast cancer relapse.

## INTRODUCTION

Breast cancer mortality is mainly associated with the development of metastases [1, 2]. The introduction of targeted therapies and optimized combinations of chemotherapeutics, coupled with better surveillance, have increased survival, especially for patients with estrogen receptor (ER) and progesterone receptor (PR) or human epidermal growth factor receptor 2 (HER2) positive primary breast cancer; however, the significance of these therapeutic and technological advances for metastatic breast cancer (MBC) survival remains controversial [2–6]. Conventionally, risk assessment and treatment decision-making for MBC is based on both clinical (length of metastasis-free interval, adjuvant systemic therapy, tumor burden, age at diagnosis, and the patient's general performance status) and primary tumor pathological factors (*i.e*. ER and HER2) [1, 2]. Typically, distant metastases have not been systematically biopsied, rationalizing the personalization of therapy based on primary tumor biomarker status. However, remarkable heterogeneity in the mutational spectrum, copy number alterations, transcriptomes and epigenomes within and between primary tumors, circulating and disseminated tumor cells and metastases have been revealed through high-throughput molecular profiling studies, and the notion that hormone receptor and HER2 status may differ significantly between metastases and primary tumors has been validated in several studies [7–12]. In addition, biomarker conversion may affect the choice of systemic therapy [11–14] and loss of ER expression at relapse has been linked with an inferior survival [9, 10]. Thus, many national and international guidelines for MBC management now recommend re-testing of at least one metastatic biopsy for better individualization of therapy [15–18]. Studies evaluating the prognostic significance of biomarker expression in metastases are of importance, especially in an era when re-testing of metastases is being mandated.

The molecular taxonomy of breast cancer is complex and currently at least four stable intrinsic subtypes with distinct biology and clinical outcome have been defined by transcriptional profiling [19–22]. However, translation of the intrinsic subtypes into daily clinical practice is limited by the requirement for fresh frozen specimens, technological complexity and high costs, and has led to the adoption of an immunohistochemistry-based surrogate definition for approximation of the intrinsic subtypes [23, 24]. The prognostic importance of the subtypes is well established in early stage disease, and they are now included in several clinical guidelines (*e.g*. St Gallen and ASCO). In contrast, the importance of the molecular subtypes is poorly defined in MBC. Hormone receptor positive tumors are comprised of two distinct subtypes; luminal A and luminal B, which differ in terms of proliferation, response to therapy and outcome. Even when ER is concordantly expressed during disease progression, a biological change in for instance proliferation, as captured by the subtypes, may occur in a metastasis, which may alter the prognosis and necessitate a change in the therapeutic management.

This study aimed to evaluate the concurrence in the expression of individual breast tumor pathological biomarkers and the tumor molecular subtypes measured at time of primary tumor diagnosis, including synchronous lymph node metastases (LNMs), and at recurrence. In addition, we aimed to investigate the significance of metastasis-specific biomarker status and the implications of biomarker conversion for the prognosis of MBC.

## RESULTS

The distribution of baseline clinico-pathological factors in the entire cohort and the subgroups of patients included in the analyses representing each tumor progression stage in this study are shown in Table 1 and Supplementary Table S1, respectively. Approximately 70% of the patients were diagnosed with synchronous LNMs. Most clinico-pathological characteristics were similarly distributed between these subsets of patients compared to the 304 patients in the study cohort (Supplementary Table S1). PR expression was however more frequently absent in the LNM metastases. Likewise, the surrogate (IHC-derived) molecular subtypes were similarly distributed between the primary tumors and the LNMs. The distribution of ER, PR and HER2 in the asynchronous metastases was similar to the primary tumor and LNM subsets, but an increase in the proportions of basal-like and HER2-enriched cases was observed among asynchronous metastases processed by transcriptional profiling (Table 1).

**Table 1:** Distribution of tumor biomarkers and molecular subtypes at different tumor progression stages

	Tumor progression stage		
	Primary Tumors (*N* = 217)	Synchronous LNM (*N* = 111)	Asynchronous Mets (*N* = 304)
	*N* (%)	*N* (%)	*N* (%)
**ER status**			
Positive	158 (81%)	75 (73%)	100 (71%)*
Negative	36 (19%)	28 (27%)	41 (29%)*
Missing/unknown	23	8	163
**PR status**			
Positive	110 (58%)	39 (38%)	53 (41%)*
Negative	81 (42%)	64 (62%)	77 (49%)*
Missing/unknown	26	8	174
**HER status**			
Amplified	17 (9%)	13 (14%)	7 (7%)*
Normal	180 (91%)	77(86%)	95 (93%)*
Missing/unknown	20	21	202
**Ki67 status**			
High	65 (36%)	33 (33%)	n.a.
Low	122 (64%)	66 (67%)	n.a.
Missing/unknown	30	12	n.a.
**Molecular subtype**			
Luminal A-like^a^/Luminal A^b^	62 (35%)^a^	25 (29%)^a^	5 (6%)^b^
Luminal B-like^a^/Luminal B^b^	84 (50%)^a^	44 (50%)^a^	26 (31%)^b^
HER2 driven^a^/HER2-enriched^b^	9 (5%)^a^	5 (6%)^a^	27 (32%)^b^
Triple negative^a^/Basal-like^b^	24 (13%)^a^	13 (15%)^a^	24 (29%)^b^
Normal-like^b^	n.a.	n.a.	2 (2%)^b^
Unclassified/missing	38	24	210

* IHC or CISH data retrieved from clinical records

a St Gallen subtype

b PAM50 subtype; n.a., not applicable

### Concordance/discordance of individual biomarkers across tumor progression stages: ER, PR, HER2 and Ki67

The expression of each biomarker was compared across different progression stages (Table 2). A total of 126 cases had paired data for ER expression between primary tumors and asynchronous metastases and a discordance rate of 17% was observed. This discordance was significantly skewed (McNemar's *P* = 0.007) as the majority (17/21, 81%) changed from positive status in the primary tumor to negative in the metastasis. Similarly, ER was discordantly expressed between 14% of the paired primary tumors and synchronous LNMs, with the majority (10/13, 77%) losing expression in the LNM (McNemar's *P* = 0.09). In contrast, 12/52 (23%) paired cases had discordant ER expression between LNMs and asynchronous metastases, but no skewness in either direction was observed as half (6/12) of the discordant cases lost ER and the other half gained ER (McNemar's *P* = 1.0).

**Table 2:** Biomarker discordance at different stages of tumor progression

Biomarker	*N*	Loss	Gain	Discordance (%)	*P*
**Primary tumor *vs*. synchronous LNM**					
ER	94	10	3	14	0.09
PR	92	16	3	21	0.004
HER2	83	2	5	8	0.45
Ki67	90	11	15	29	0.56
**Primary tumor *vs*. asynchronous metastasis**					
ER	126	17	4	17	0.007
PR	105	32	9	39	<0.001
HER2	64	1	0	2	1.0
**Synchronous LNM *vs*. asynchronous metastasis**					
ER	52	6	6	23	1.0
PR	44	10	8	41	0.82
HER2	30	3	0	10	0.25

Abbreviations: *N*, number of cases with paired data; *P*, *P*-value (McNemar's test)

PR expression was more unstable between the different tumor progression stages. Discordance rates of 21% and 39% were observed between primary tumors and LNMs, and between primary tumors and asynchronous metastases, respectively (Table 2). A significant skewness from positive to negative status (16/19, 84%) was observed for primary tumors *vs*. LNMs (McNemar's *P* = 0.004) and similarly, 32/41 (78%) discordant cases lost PR expression when primary tumors were compared with paired asynchronous metastases (McNemar's *P* < 0.001). No statistically significant bias in the direction of change was noted for LNMs compared with asynchronous metastases. Although 18/44 (41%) paired cases were discordant, only 10/18 (56%) lost PR expression (McNemar's *P* = 0.82).

Discordance rates for HER2 and Ki67 expression between primary tumors and LNMs were 8% and 29%, respectively (Table 2). Five of seven (71%) discordant cases gained HER2 expression, while 15/26 (58%) of cases with discordant Ki67 displayed high expression of this proliferation marker in the LNM. However, no statistically significant skewness in the direction of change was observed for these biomarkers (McNemar's *P* > 0.05).

### Contrasting molecular subtypes across tumor progression stages

The overall distribution of molecular subtypes was similar between primary tumors and synchronous metastases, but there was an enrichment for more aggressive subtypes (HER2-enriched and basal-like) in the asynchronous metastases (Table 1). Surrogate molecular subtypes could be assigned to 179 primary tumors and 87 LNMs respectively, of which 74 cases had paired data. A subtype conversion was observed in 13/33 (39%) patients with luminal A-like primary tumors, 92% (12/13) of which changed to the luminal B-like subtype. In contrast, a luminal B-like subtype in the primary tumor changed to the less aggressive luminal A-like subtype in 5/28 (18%) patients, and to the more aggressive triple negative subtype in 2/28 (7%) patients. Three of nine (33%) triple negative primary tumors also changed subtype. The McNemar-Bowker's test of symmetry was used to test the null hypothesis that the shift in the distribution of subtypes was balanced, resulting in no significant deviation from the null hypothesis (Table 3; McNemar-Bowker's *P* = 0.42).

**Table 3:** Breast cancer molecular subtype concordance/discordance at different stages of tumor progression

**LNM (St Gallen subtype)**					
**Primary tumor (St Gallen subtype)**	Luminal A-like	Luminal B-like	HER2 driven	Triple negative	P*
Luminal A-like	20	12	0	1	0.42
Luminal B-like	5	21	0	2	
HER2 driven	0	0	3	1	
Triple negative	0	2	1	6	
**Asynchronous metastasis (PAM50 subtype)**					
**Primary tumor (St Gallen subtype)**	Luminal A	Luminal B	HER2-enriched	Basal-like	P*
Luminal A-like	2	8	3	0	0.001
Luminal B-like	1	7	11	2	
HER2 driven	0	1	3	1	
Triple negative	0	0	0	10	
**Asynchronous metastasis (PAM50 subtype)**					
**LNM (St Gallen subtype)**	Luminal A	Luminal B	HER2-enriched	Basal-like	P*
Luminal A-like	0	3	2	0	0.09
Luminal B-like	2	8	4	2	
HER2 driven	0	0	2	0	
Triple negative	0	0	0	6	

* *P*-value from McNemar-Bowker's test.

Next, an exploratory analysis was performed to compare the molecular subtypes between primary tumors and asynchronous metastases. To simplify the analyses, the triple negative and basal-like subgroups were considered as the same entity since the basal-like subtype is known to constitute a majority of the triple negative group [22, 25, 26]. Furthermore, because the surrogate IHC classification does not include the normal-like subgroup, the two metastases assigned to this subgroup by PAM50 were excluded. In total, 49 cases with paired data for the molecular subtypes from the primary tumors and asynchronous metastases were included in the final analyses (Table 3). Eleven out of 13 (85%) cases changed subtype from luminal A-like in the primary tumor to a more aggressive subtype in the metastasis [luminal B (8/11, 73%) and HER2-enriched (3/11, 27%), respectively]. In addition, 14/21 (67%) cases transitioned from the luminal B-like subtype, 11 (79%) switching to the HER2-enriched subtype. Of note, the majority (3/5, 60%) of the HER2 driven primary tumors were of the HER2-enriched subtype at recurrence and all (10/10, 100%) triple negative primary tumors displayed a basal-like subtype at recurrence. Overall, the symmetry of subtype distribution between the primary tumors and asynchronous metastases was significantly altered, particularly for the luminal tumors, favoring a shift to a more aggressive subtype at recurrence (11/11 for luminal A-like and 13/14 for luminal B-like (Table 3; McNemar-Bowker's *P* = 0.001). Similarly, a very high (100%) concordance of the HER2 driven/HER2-enriched and triple negative/basal subtypes was observed when LNMs and asynchronous metastases were contrasted. All (5/5) luminal A-like tumors also changed subtype in the asynchronous metastasis, while 6/10 luminal B-like tumors changed to a more aggressive subtype (Table 3; McNemar-Bowker's *P* = 0.09).

Additional analyses were performed to investigate possible associations between biomarker conversion and the administration of adjuvant treatment for primary disease (endocrine or chemotherapy treatment), as well as the association between biomarker drifts and the site/s of metastatic recurrence. However, no significant association was observed for any of these comparisons (Fisher's exact *P* > 0.05 for all tests). In addition, subtype conversion was not associated with time to progression after primary tumor diagnosis (Log-rank *P* = 0.52).

### Post-recurrence breast cancer mortality in relation to ER, PR and molecular subtype status at different progression stages

**Figure 1:**
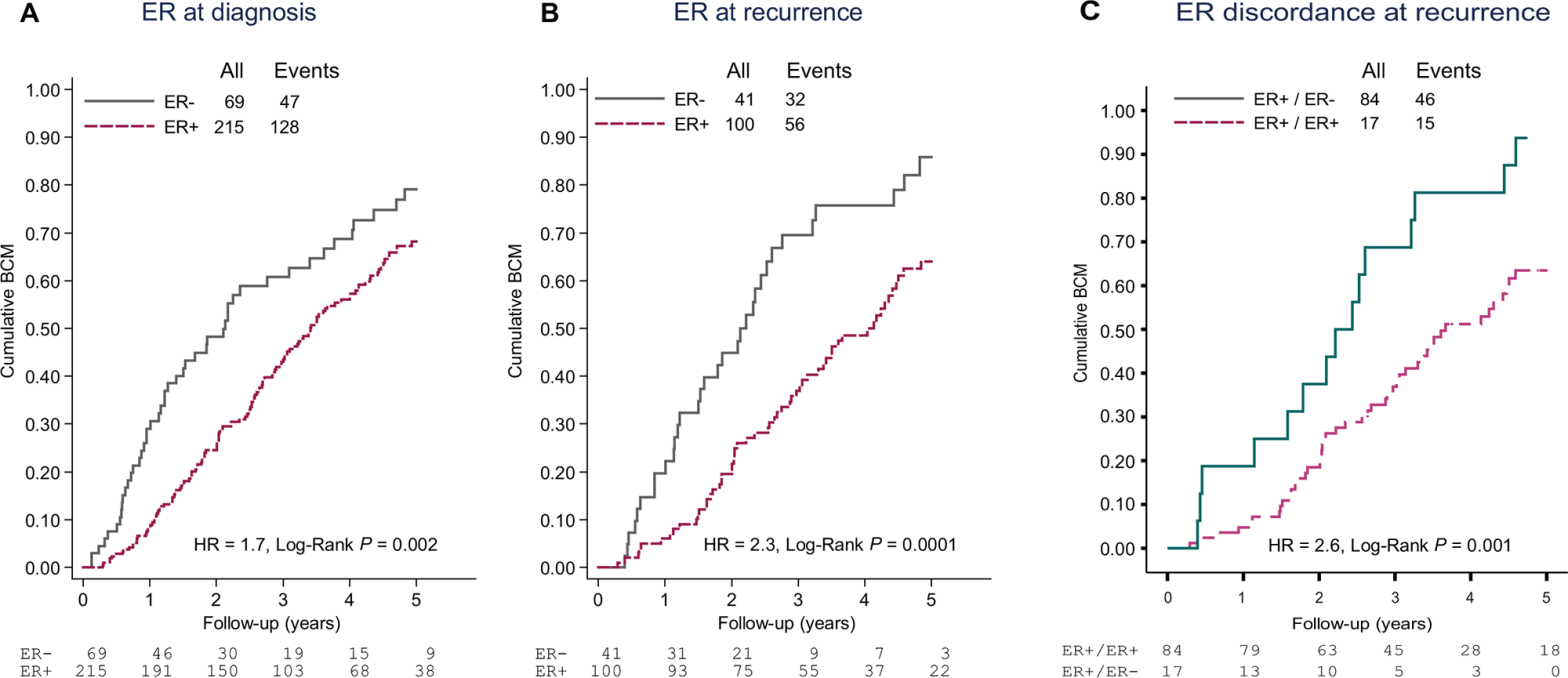
Cumulative breast cancer mortality (BCM) following metastasis diagnosis according to A. ER status at primary diagnosis, B. ER status at recurrence and C. Discordance in ER expression between primary tumor and asynchronous metastasis Abbreviations: ER+, estrogen receptor positive; ER-, estrogen receptor negative.

The median breast cancer specific survival for patients alive at last follow-up was approximately 45 months (range 9-135 months). Negative ER status at primary diagnosis was associated with inferior survival in both univariable (primary tumors; Figure 1A, Log-rank *P* = 0.002, HR = 1.7, CI = 1.3–2.4 and LNMs; HR = 2.1, CI = 1.3–3.5) and multivariable analyses (Table 4: primary tumors; HR = 2.2, CI = 1.5–3.3 and LNMs; HR = 2.0, CI = 1.2–3.3). Interestingly, the relative risk of mortality in the ER negative group increased when the ER status of the asynchronous metastases was considered [univariable (Figure 1B, Log-rank *P* = 0.0001 HR = 2.3, CI = 1.5–3.6) and multivariable analyses (Table 4; HR = 3.0, CI = 1.8–5.0)]. Negative PR status in the primary tumors and asynchronous metastases only was also independently prognostic for shorter survival following relapse in multivariable analyses (Table 4: primary tumors; HR = 1.5, CI = 1.1–2.1 and asynchronous metastases; HR = 2.0, CI = 1.2–3.4).

**Table 4:** Multivariable Cox proportional hazards analyses for 5-year post-recurrence breast cancer mortality (BCM)

	*N*	Relative hazard	95% CI	*P*
**Biomarker status of primary tumors**				
**ER**	275			
Positive (reference)	210	1.0		
Negative *vs*. positive	65	2.2	1.5–3.3	<0.001
**PR**	259			
Positive (reference)	149	1.0		
Negative *vs*. positive	110	1.5	1.1–2.1	0.02
**St Gallen subtype**	175			0.003
Luminal A-like (reference)	64	1.0		
Luminal B-like *vs*. Luminal A-like	81	1.3	0.82–1.9	0.31
HER2 driven *vs.* Luminal A-like	8	2.5	1.0–5.8	0.04
Triple negative *vs*. Luminal A-like	23	3.1	1.7–5.9	<0.001
**Biomarker status of LNM**				
**ER**	103			
Positive (reference)	75	1.0		
Negative *vs*. positive	28	2.0	1.2–3.3	0.01
**PR**	103			
Positive (reference)	39	1.0		
Negative *vs*. positive	64	0.99	0.59–1.66	0.97
**St Gallen subtype**	87			<0.001
Luminal A-like (reference)	25	1.0		
Luminal B-like *vs*. Luminal A-like	44	0.86	0.45–1.7	0.66
HER2 driven *vs.* Luminal A-like	5	0.56	0.18–1.8	0.32
Triple negative *vs*. Luminal A-like	13	7.9	3.2–19.5	<0.001
**Biomarker status of asynchronous metastases**				
**ER**	135			
Positive (reference)	95	1.0		
Negative *vs*. positive	40	3.0	1.8–5.0	<0.001
**PR**	125			
Positive (reference)	50			
Negative *vs*. positive	75	2.0	1.2–3.4	0.01
**PAM50 subtype**	78			0.018
Luminal A (reference)	5	1.0		
Luminal B *vs*. Luminal A	26	4.4	0.51–36.8	0.18
HER2-enriched *vs*. Luminal A	24	8.1	0.93–70.1	0.06
Basal-like *vs*. Luminal A	23	17.3	1.7–176.7	0.016

The analyses were adjusted for age at primary diagnosis (>50 years or ≤50 years), metastasis-free interval (≤2 years or >2 years), number of metastatic sites (multiple or single), site of metastasis (loco-regional *vs*. bone or lung or liver), nodal status (N+ or N0), adjuvant endocrine therapy (yes or no), and adjuvant chemotherapy (yes or no)

**Figure 2:**
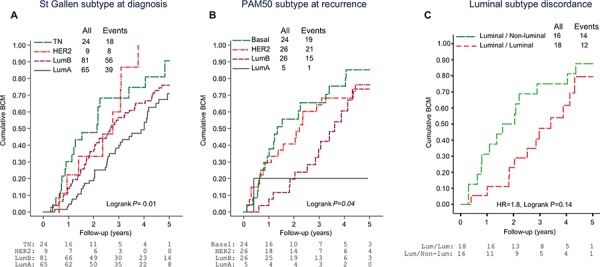
Cumulative breast cancer mortality (BCM) following metastasis diagnosis according to A. St Gallen molecular subtype at primary diagnosis, B. PAM50 molecular subtype at recurrence and C. Conversion from a luminal-like to a non-luminal subtype between primary tumor and asynchronous metastasis Abbreviations: Lum, Luminal; LumA, Luminal A; LumB, Luminal B; HER2, HER2-enriched; TN, Triple negative.

Next, the prognostic significance of the molecular subtypes for outcome after MBC diagnosis was investigated. Five-year post-relapse mortality rates were significantly different between the molecular subtypes assessed in primary tumors (Figure 2A; Log-rank *P* = 0.01) and synchronous LNMs (Log-rank *P* < 0.001) and subtype remained an independent prognostic factor in multivariable analyses (Table 4; *P* < 0.01). Importantly, a significant difference in post-recurrence breast cancer mortality was also observed when the PAM50 intrinsic subtypes of the metastases were considered [univariable (Figure 2B; Log-rank *P* = 0.04) and multivariable (Table 4; *P* = 0.02) analyses].

Finally, the effect of losing ER expression at recurrence or changing tumor subtype from a luminal-like subtype in the primary tumor to a non-luminal subtype in the asynchronous metastases was investigated in sub-analyses. Amongst patients presenting with ER positive primary tumors and for whom paired data for ER expression in the asynchronous metastases could be retrieved from the clinical records (*N* = 101), 17 (17%) lost ER expression at recurrence, which conveyed a significantly inferior breast cancer survival (Figure 1C, HR = 2.6, CI = 1.5–4.7, *P* = 0.002). Similarly, when considering only patients with a luminal-like (luminal A or luminal B) primary tumor, 16/34 cases changed to a non-luminal subtype following adjuvant treatment. The majority (14/16) became HER2-enriched, and this drift in molecular subtype correlated with an inferior post-recurrence survival (Figure 2C, HR = 1.8, CI = 0.82–3.9, *P* = 0.14). Interestingly, amongst these 16 cases, where corresponding IHC or CISH data for ER and HER2, respectively were available, 7/10 had concordant ER positive tumors and only 1/7 had gained HER2 amplification in the metastasis.

## DISCUSSION

This study confirms that conversion of single tumor pathological biomarkers occurs continuously during the course of breast cancer progression, with loss of hormone receptors (ER and PR) most commonly observed when primary tumor cells migrate from the breast to other anatomical sites. In addition, we confirm that loss of ER expression following adjuvant treatment is significantly associated with an unfavorable post-recurrence survival. Importantly, our results also suggest that other tumor biological properties which are captured when evaluating the molecular subtypes may also change in metastases, with important consequences on post-recurrence disease outcome. Such important changes in tumor biology may be missed when only single biomarkers are evaluated, as evident by the drifts observed in ER positive primary tumors even when ER is concordantly expressed in the metastasis. The implication of these results for the management of MBC is important given the poor prognosis generally associated with an MBC diagnosis.

In this study, 17% and 39% discordance rates were observed for ER and PR expression respectively, between primary tumors and asynchronous metastases, which is consistent with the 7–31% and 21–49% discordance rates reported for ER and PR respectively in previous studies [7, 8, 10, 12, 27–29]. Similarly, ER and PR expression was also unstable when primary tumors were contrasted with synchronous LNMs, albeit to a lesser extent. A similar comparison by Falck et al. [30] did not reveal any statistically significant changes in ER or PR expression between primary tumors and synchronous LNMs, but the smaller number of cases included in their analyses and the enrichment for patients with metastatic breast cancer in the present cohort may have contributed to the divergent results. The presence of synchronous LNMs at time of primary tumor diagnosis is prognostic for a higher likelihood of relapse and death due to breast cancer [1, 2], yet their contribution to the biology of metastatic disease is not well studied. Although the level of discordance for ER and PR between LNMs and asynchronous metastases was comparable to that between primary tumors and asynchronous metastases, the direction of change was not significantly skewed in either direction, probably due to the smaller number of cases included in these analyses. Notwithstanding, these data highlight the vast intra- and inter-tumoral heterogeneity between primary tumors and metastases, which represents a severe impediment to the successful clinical management of breast cancer.

ER is an acknowledged independent prognostic and treatment predictive factor in primary breast cancer and the intrinsic molecular subtypes have also been validated for prognostication purposes in early stage disease [31]. However, unlike the hormone receptors, the role of the molecular subtypes is not well defined in MBC. Primary tumors of the triple negative and HER2 positive subtypes were remarkably stable throughout tumor progression. On the other hand, luminal-like tumors were significantly unstable, frequently adopting a more aggressive and proliferative phenotype in the metastases. Importantly, the fact that a majority of the cases with discordant luminal subtypes still expressed ER highlights the importance of determining the intrinsic molecular subtype in addition to ER in metastases. Analogous to our findings, Falck *et al*., by using the IHC-based classification as outlined in the 12^th^ St Gallen consensus guidelines [24], reported more frequent, albeit statistically non-significant, conversion from luminal A to luminal B in LNMs and luminal A to luminal B/HER2 positive at recurrence [30]. Patients with metastases displaying a conversion from a luminal-like to a HER2-enriched subtype may derive benefit from treatment with anti-HER2 agents. Nonetheless, given the small number of cases included in our study and that of Falck and colleagues [30], further investigations are warranted to validate and extend these interesting results.

Currently, the prognosis of MBC is established based on primary tumor characteristics, including ER and HER2 status. Although ER status of the primary tumor has been validated as an independent prognostic factor for post-recurrence survival [1], such analyses ignore the possibility of a conversion of the ER status in the metastasis and the consequences there-of. In separate Cox-proportional multivariable models, ER and molecular subtype at time of primary diagnosis and at recurrence were both independent prognostic factors of breast cancer mortality; but interestingly, mortality risk estimates were higher when biomarkers assessed at time of relapse were modeled. However, because of unequal numbers of cases included in the different models, statistical tests to evaluate the best performing model were not performed. Nonetheless, our results confirm the validity of ER and molecular subtypes of both the primary tumor and metastasis as independent prognostic biomarkers for clinical outcome after breast cancer relapse.

Biopsies of metastases are now routinely collected whenever possible for reassessment of biomarkers, but data on the impact of biomarker discordance on decisions regarding treatment choice and overall survival are scarce. We show that loss of ER at recurrence is associated with an inferior prognosis, a finding in line with previous reports [9, 10, 29, 30]. Furthermore, conversion from a luminal to a non-luminal subtype at recurrence was also associated with shorter post-recurrence survival. Together, these findings link biomarker discordance with changes in tumor biology due to selective pressures of adjuvant systemic treatment, resulting in more aggressive metastatic clones which may require alteration of treatment strategies to improve survival following breast cancer recurrence.

Despite the significant associations of drifts in biomarker expression and clinical outcome shown here-in, these discordances may also be attributed to less-than-perfect accuracy and reproducibility of analytical techniques [9, 15, 16, 32] and specific to this study, differences in the methods used for assigning subtypes to the primary tumors (IHC) and the asynchronous metastases (mRNA profiles). The threshold used to define ER positivity may affect concordance rates [7]. Although the locally accepted 10% cut-off for ER positivity used in this study differs from the 1% cut-off recently recommended by the ASCO/CAP guidelines [33], a sub-analysis using the 1% cut-off for ER showed similar results, precluding any potential influence of ER cut-off on the results reported. PR, as expected, displayed the highest rates of change across tumor progression stages and could in part explain the high rate of conversion from luminal A-like to luminal B at recurrence. The compatibility between the IHC-based subtype classification and the gene expression-based PAM50 classification is modest [21, 26, 34, 35], and the expected discordance rate associated with analytical bias has been shown to range between 15–19% [26, 34] for the luminal A subtype, which is significantly lower than the 85% discordance observed in the present study. One would also expect a more frequent misclassification of luminal-like tumors with amplification of the HER2 gene (ER+, HER2+, any PR, any Ki67) to HER2-enriched by PAM50 [21, 26, 34, 35]. Importantly in the present study, almost all the luminal-like to HER2-enriched subtype conversion occurred in tumors lacking HER2 amplification, since HER2 positivity was an exclusion criteria in the clinical trial on which the present study was based. In fact, the two cases with HER2 positive primary tumors with corresponding PAM50 subtype for the asynchronous metastases were classified as HER2-enriched by PAM50, as expected. Taken together, these data suggest that some ER positive tumors may adopt a HER2-enriched phenotype at recurrence in the absence of an *ERBB2* amplification, a change that may only be captured by subtyping using gene expression methods such as PAM50. Furthermore, a true subtype conversion may have occurred in a significant proportion of these discordant cases based on the significant adverse correlation with prognosis observed. The triple negative and basal subtypes are reported to display the highest concordance [21, 26, 35] which was also observed in our study, further validating these reports. Clearly, given the importance of translating the intrinsic subtypes into clinically useful groups, there is an urgent need for prospective data to strengthen the clinical validity and utility of individual biomarkers, especially cut-offs for Ki67 and PR, to optimize and standardize the methodologies for the identification of molecular subtypes whether by IHC or mRNA profiling.

In summary, receptor conversion and change in molecular subtype at recurrence can potentially affect the outcome of patients diagnosed with MBC, and biomarker assessment at time of recurrence may significantly improve prognostication and treatment of MBC, as evidenced by data presented here-in. However, some caveats of our study call for caution when interpreting the significance of these results. First, the retrospective nature of this study limited the sample size in several analyses. In addition, as a result of limited resources, a technical bias due to methodological differences in the assessment of molecular subtypes in primary tumors and asynchronous metastases may have affected the results, and the potential effects of intra-tumoral heterogeneity on biomarker evaluation warrants further prospective studies to better understand the significance of these interesting results given their clinical value to improve the personalization of therapy for women diagnosed with MBC.

## MATERIALS AND METHODS

### Patients

The study cohort consisted of 304 women with locally advanced and MBC who were enrolled in a randomized phase III trial (TEX) conducted between 2002–2007 in Sweden to study the effects of two first-line chemotherapy regimens for MBC. Patients with brain metastases, HER2-positive disease indicated for first-line treatment with trastuzumab, or with other malignancies diagnosed within five years were exempted from the trial. Detailed information on the trial design and outcome has been reported [36]. As first-line treatment for MBC, patients received a combination of an anthracycline (epirubucin) and a taxane (paclitaxel) with or without the addition of capecitabine; no significant difference in overall survival was observed between the treatment arms.

### Ethics statement

This sub-study was approved by the regional ethics committees at all participating centers: Karolinska Institutet Stockholm (KI 02–205 & 02–206); Sahlgrenska University Hospital Gothenburg (M090–02 & M091–02); Linköping University Hospital (02–519 & 02–339); Örebro University Hospital (308/02 & 308/03); Umeå University Hospital (Um 02–336 & Um 03–03) and Lund University Hospital (LU 290–02 & LU 291–02). All patients provided written informed consent to participate in the clinical trial and translational studies, including genomic analyses and publication of results (ClinicalTrials.gov Identifier: NCT01433614). This study adheres to the REMARK guidelines for reporting tumor marker prognostic studies [37].

### Data collection

The flow chart in ww depicts patient/sample selection at the respective stages. Pathological data, adjuvant systemic treatment and outcome data were collected from the central clinical trial database. Archival formalin-fixed paraffin-embedded primary tumor and LNM blocks were collected whenever possible for tissue microarray (TMA) construction and central assessment of biomarkers by IHC. Cases were excluded from analyses if TMA core loss occurred during IHC staining procedures, non-evaluable staining, <10% tumor cells available for scoring or if clinical data were missing.

Furthermore, fine-needle aspirates (FNAs) from metastatic lesions were collected for transcriptional profiling whenever possible as previously described [39]. In brief, tumor cellularity was assessed by a cytologist and total RNA was extracted from samples with high (>50%) tumor cell content. RNA quantity and integrity were analyzed using the NanoDrop spectrophotometer (NanoDrop Technologies, Wilmington, DE) and the Agilent 2100 Bioanalyzer (Agilent, Santa Clara, CA). Samples with sufficient high quality RNA were hybridized onto custom-made Affymetrix HuRSTA-2a520709 gene chips. Data pre-processing and normalization were performed using the robust multichip average (RMA) algorithm. Of the 120 metastases collected, 92 samples obtained from 85 patients passed all quality control measures and were included in the final dataset. These data can be accessed from the Gene Expression Omnibus database (GSE46141).

### Biomarker evaluation

**Figure 3:**
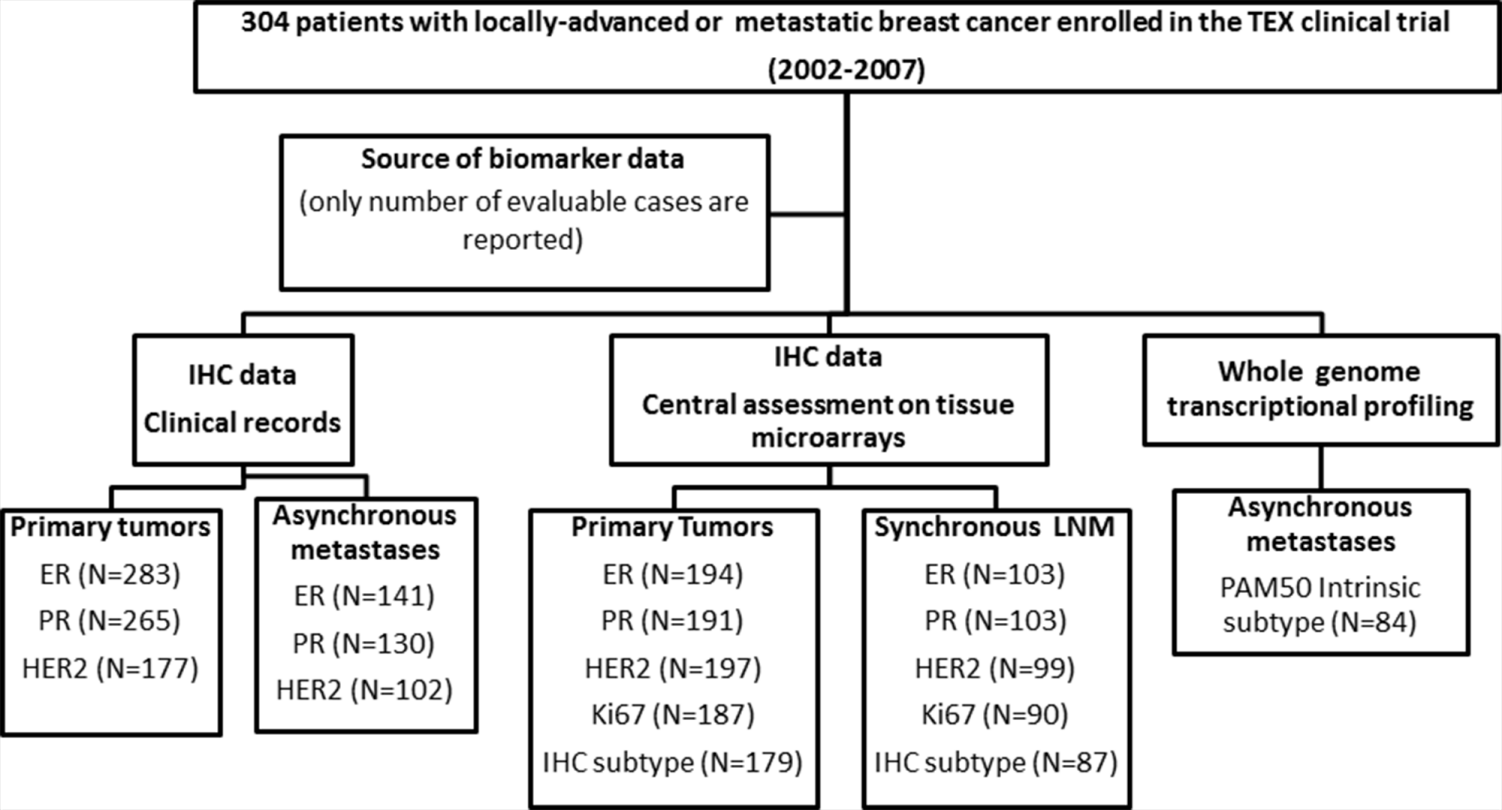
Flow chart showing the selection of patients included at different tumor progression stages Cases were excluded due to missing clinical data, unavailable tumor blocks, missing TMA data due to core loss or <10% tumor cells, or failed quality control for transcriptional profiling, respectively. Abbreviations: ER, estrogen receptor; PR, progesterone receptor; HER2, human epidermal growth factor receptor-2; IHC, immumohistochemistry.

Standard pathological markers (ER, PR, HER2 and Ki67) were centrally evaluated on the TMAs. At least two 0.6 mm cores per sample were included on the TMAs. This number of cores has been reported to be sufficient to produce results representative of staining seen on full histological sections, even for markers that are heterogeneously expressed [11, 38]. The complete information describing TMA construction and evaluation of biomarkers has been previously reported [39]. All scorings were performed independently by a pathologist blinded to outcome information. Briefly, two representative cores from the invasive component of the tumor were taken from the donor FFPE block to the TMA. ER, PR and Ki67 were analyzed by IHC and HER2 status was analyzed by both IHC and chromogenic *in situ* hybridization (CISH). Furthermore, IHC data for ER, PR and HER2 (performed on core or fine-needle biopsies) for asynchronous metastases were extracted from the clinical trial database. Cut-offs used to indicate positive staining were ≥10% for ER and PR, IHC 3+ or positive CISH for HER2 and ≥20% for Ki67, as recommended by national guidelines. For determination of the surrogate molecular subtype of tumors, a cut-off of ≥20% was used to indicate PR positivity as recommended by the 2013^th^ St Gallen consensus guidelines [23]. In addition, IHC/CISH data for ER, PR and HER2 for asynchronous metastases were retrieved from clinical records whenever possible. The final number of evaluable cases for each biomarker is presented in Figure 3 and Table 1.

### Molecular subtyping

The St Gallen International experts’ consensus on primary therapy for early breast cancer approved the utilization of a surrogate molecular subtype classification for breast cancers based on IHC assessment of ER, PR, HER2 and Ki67 [23, 24]. Following the guidelines from the 2013 experts’ consensus [23], tumors were classified into the following molecular subgroups: luminal A-like (ER+, PR+, HER2-, Ki67 low), luminal B-like (ER+, HER2- and at least one of PR- or Ki67 high; or alternatively ER+, HER2+, any PR, any Ki67), HER2 driven (ER-, PR-, HER2+, any Ki67) and triple negative (ER-, PR-, HER2-, any Ki67). In addition, the PAM50 genetic classifier was used for classifying metastases into intrinsic subtypes (luminal A, luminal B, HER2-enriched, basal-like and normal-like) as originally described [21]. For accurate determination of the intrinsic subtype of a tumor, the sample must be centered against an appropriately large and representative sample set. HER2 positive disease was a basis for exclusion from the TEX trial, hence the distribution of the subtypes in the present cohort may be different from that of the original dataset used by Parker and colleagues [21], and may violate the specifications for accurate assignment of subtypes using the PAM50 classifier. To circumvent this potential bias, the breast cancer metastasis dataset used in the present investigation was merged with an external primary breast cancer dataset (*N* = 623, GSE48091) profiled using the same microarray platform (Affymetrix HuRSTA-2a520709 gene chips) to obtain a sufficiently sized and representative cohort to improve the accuracy in intrinsic subtype determination. The data were normalized and classified into the intrinsic subtypes as previously described [21].

### Statistical analyses

Paired dichotomized data were compared using the McNemar test. McNemar-Bowker's test of symmetry was used when comparing molecular subtypes between tumor progression stages. The primary endpoint for survival analyses was post-recurrence breast cancer mortality (BCM), which was calculated as the interval between the diagnosis of metastatic disease to breast cancer specific death, and the follow-up was restricted to five years. The last follow-up date was July of 2013 and the median post-recurrence BCM was approximately 2.7 years. Cumulative incidence curves were used to visualize differences in BCM between groups and the Log-rank test to evaluate statistical significance. Univariable and multivariable Cox-proportional hazards models were used to evaluate the independent prognostic importance of biomarkers and molecular subtype. Proportional hazards assumptions were checked by graphical methods. All *P*-values correspond to two-sided statistical tests and values <0.05 were considered significant. The statistical software packages IBM SPSS Statistics 19 (IBM Corporation, NY) and STATA version 12 (StataCorp, College Station, TX) were used.

## SUPPLEMENTARY TABLE


