# Supplementary material for: Contrasting breast cancer molecular subtypes across serial tumor progression stages: biological and prognostic implications
Source: Oncotarget. 2015 Sep 8;6(32):33306–18. doi: 10.18632/oncotarget.5089 (PMC4741767; doi:10.18632/oncotarget.5089)
Supplement: Supplementary file 1 [file oncotarget-06-33306-s001.pdf]

SUPPLEMENTARY TABLE

Supplementary Table S1: Baseline clinical and pathological characteristics of patients and primary tumors for the entire study cohort and the subsets of patients included on the two different tissue microarrays (TMAs)

		Tissue Microarrays (TMAs)	
	All Patients (*N* = 304)	Primary Tumors (*N* = 217)	Synchronous LNMs (*N* = 111)
	N (%)	*N* (%)	*N* (%)
**Age at diagnosis**			
Median (range)	50 (27–71)	51 (27–71)	51 (27–71)
< 50 years	154 (51%)	97 (45%)	52 (42%)
≥ 50 years	149 (49%)	118 (55%)	72 (58%)
Missing/unknown	1	2	0
**Histological grade**			
1/2	121 (49%)	80 (43%)	39 (43%)
3	125 (51%)	105 (57%)	52 (57%)
Missing/unknown	58	32	20
**Tumor size**			
≤ 20 mm	119 (40%)	84 (40%)	31 (28%)
> 20 mm	180 (60%)	128 (60%)	79 (72%)
Missing/unknown	5	5	1
**Nodal status**			
N0	92 (31%)	68 (33%)	0 (0%)
N+	203 (69%)	141 (67%)	111 (100%)
Missing/unknown	9	8	0
**Adjuvant chemotherapy**			
Yes	150 (49%)	101 (47%)	72 (65%)
No	152 (50%)	114 (53%)	39(35%)
Missing/unknown	2	2	0
**Adjuvant endocrine therapy**			
Yes	156 (52%)	117 (54%)	70 (63%)
No	147 (48%)	99 (46%)	41 (37%)
Missing/unknown	1	1	0
**Adjuvant radiotherapy**			
Yes	214 (71%)	154 (72%)	84 (76%)
No	87 (29%)	60 (28%)	26 (24%)
Missing/unknown	3	3	1

(*Continued*)

		Tissue Microarrays (TMAs)	
	**All Patients (*N* = 304)**	**Primary Tumors (*N* = 217)**	**Synchronous LNMs (*N* = 111)**
	***N* (%)**	***N* (%)**	***N* (%)**
**Metastasis-free interval**			
Locally-advanced	47 (16%)	14 (7%)	15 (13%)
< 5 years	147 (48%)	124 (57%)	66 (60%)
≥ 5 years	110 (36%)	79 (36%)	30 (27%)
Missing/unknown	0	0	0
**Site of recurrence**			
Loco-regional	50 (17%)	38 (18%)	19 (17%)
Bone	56 (18%)	38 (18%)	21 (19%)
Lung	63 (21%)	45 (21%)	21 (19%)
Liver	133 (44%)	94 (43%)	50 (45%)
Missing/unknown	2	1	
